# Application of improved ant-lion algorithm for power systems

**DOI:** 10.1371/journal.pone.0311563

**Published:** 2024-12-04

**Authors:** Wenjing Wang, Renjun Zhou

**Affiliations:** School of Information Engineering, Shanxi Vocational University of Engineering Science and Technology, Taiyuan, China; Shanghai Jiaotong University: Shanghai Jiao Tong University, CHINA

## Abstract

An improved ant-lion algorithm is proposed to solve the load allocation problem to improve the efficiency of load allocation in the power system. The global search capability and optimization performance of the algorithm have been significantly improved by introducing elite weights and chaotic search mechanisms. The innovation of the research lies in not only optimizing economic goals, but also considering environmental goals, achieving dual optimization of economy and environment. The average running time of the proposed algorithm in Sphere function and Griebank function was 2.67s and 1.64s, respectively. The required number of iterations was significantly better than other algorithms. In the verification of solving economic load dispatch, the improved ant-lion optimizer achieved a total fuel cost reduction of 0.10% -2.39% and 6% in both 3-unit and 6-unit simulations, respectively, compared to the other three algorithms. In the verification of solving environmental and economic load dispatch, considering the valve point effect, this proposed optimization scheme had a total fuel cost of 622.46 $/hr and a total emission of 0.20 tons/h. The total objective function was 1542.54 $/hr, which was an average reduction of 53.55 $/hr compared to the other five algorithms. Therefore, improving the ant-lion optimizer can enhance its optimization performance. The improved ant-lion optimizer has positive application significance in power system load dispatch and can achieve superior load dispatch results.

## 1. Introduction

As the proportion of new energy power in the new Power System (PS) continues to increase, the demand for load dispatch within the PS for unit peak shaving is becoming increasingly high [1]. In the normal operation of PS, it is not only necessary to ensure system safety and reliability, but also to ensure economic and environmental benefits [2]. Therefore, it is particularly necessary to improve the efficiency of Economic Load Dispatch (ELD) and Economic Emission Load Dispatch (EELD) in PS and obtain the optimal allocation scheme. ELD and EELD, as optimization problems in PS, allocate the output of each unit reasonably while ensuring power balance. This can save energy consumption and improve economic and environmental benefits [3]. ELD, EELD, and other problems have non-convex and nonlinear, high-dimensional, and non-differentiable characteristics [4]. Traditional solutions rely on classical mathematical algorithms for solving. However, in related research, the effectiveness of classical mathematical algorithms in handling ELD and EELD is not ideal [5]. The development of artificial intelligence has led to the widespread application of some intelligent optimization algorithms in various fields. Ant-Lion Optimizer (ALO), as an intelligent algorithm for solving optimization problems, imitates the intelligent behavior of ant-lions preying on ants for optimal solution. ALO has the characteristics of simple application, reliability, and superior efficiency [6]. However, due to probability mechanisms such as random walks and roulette wheel games, ALO is prone to falling into local optima and cannot guarantee global search of the solution space. Chaos Game Optimization (CGO), as an emerging metaheuristic optimization algorithm, has significant advantages in terms of search efficiency and convergence accuracy. Therefore, this study introduces CGO to improve it and applies the Improved Ant-Lion Optimizer (IALO) to deal with the ELD and EELD in PS. The aim is to improve the ELD and environmental benefits in PS.

IALO is proposed for solving multi-objective economic load allocation problems in PS. IALO improves the global search capability and optimization performance of the algorithm by introducing elite weights and chaotic search mechanism. IALO is used to solve the ELD and EELD problems, which effectively solves the optimization problems in PS and improves the economy and environmental protection of PS. The superiority of IALO in terms of fuel cost reduction and emission reduction is verified by comparing with several existing algorithms. As for the PS load allocation problem solving, the study not only considers the economic objective but also the environmental objective at the same time, which reflects the importance of sustainable development. The validation results demonstrate the application value of the proposed method in real PS optimization. IALO is able to output a more desirable load allocation result while considering Valve Point Effect (VPE).

The novelty of the study is the consideration of economic objectives along with environmental objectives, which achieves both economic and environmental optimization. The study introduces a price penalty factor to transform the EELD problem from a multi-objective optimization problem to a single-objective problem. The study provides a new way to weigh the economic and environmental impacts of PS load allocation by balancing the economic costs and emissions in the optimization process. In comparison with existing algorithms, IALO shows significant performance improvement in terms of objective function values. IALO achieves lower fuel costs and emissions in both economic load allocation and economic emission load allocation problems.

This study consists of four parts. Firstly, the research achievements and shortcomings on PS load dispatch and ALO are summarized. Secondly, the mathematical models of ELD and EELD, as well as IALO, are studied and designed. Next, IALO is adopted to verify and analyze the mathematical models of ELD and EELD through examples. Finally, these results are summarized and future research directions are indicated.

## 2. Related works

The load dispatch results affect the stable operation of PS. How to achieve reasonable, reliable, and efficient load dispatch has become a current research hotspot. W. K. Hao et al. proposed a new improved means using basic function interference to deal with ELD. A balance between algorithm exploration and utilization ability was achieved by adding elementary functions on the basis of mathematical optimization acceleration and mathematical optimization probability. This improved the algorithmic convergence speed and global search ability in solving ELD [7]. M. A. Al Betar et al. put forward a hybrid sine cosine approach in the form of memory technology to solve ELD. The optimal solution was achieved by using mathematical models of sine and cosine trigonometric functions. A climbing optimizer was introduced for local search, thus avoiding the constraints of slope and forbidden operation zone on the solution space [8]. S. Basak et al. proposed the price penalty factor approach and fractional programming approach for systematic economic emission scheduling of renewable energy clean PS. By mixing the crow search algorithm with Java as an optimization tool, the environmental pollution caused by power distribution results was reduced [9]. J. Singh et al. proposed a multi-objective adaptive encoding quantum inspired evolutionary approach. This solved the economic emission compliance scheduling for multi-objective queues such as emissions and costs. On the basis of repairing constraint processing and adaptive quantum crossover algorithm, all constraint conditions of the unit and system were satisfied. This achieved ideal optimization results in bus systems composed of different generators [10]. J. Soni and K. Bhattacharjee considered actual nonlinear constraints to lower the pollutant gas emissions. Then a multi-objective dynamic economic emission scheduling model including plug-in electric vehicles was proposed to be solved using a balance optimizer. This achieved ideal integration efficiency in different situations of thermal power generation units [11].

I. Ahmed et al. proposed a consensus-based dynamic event-triggered distributed optimization method for the Economic Dispatch (ED) problem of a population of power generating units in a communication network. This method led to a decentralized optimization solution of the total cost function while maintaining the communication efficiency [12]. M. Moin et al. proposed a new consensus-directed optimization method for the distributed power generation facility for the problem of complex nonlinear VPE. They proposed a new distributed consensus-oriented optimization method. This method achieved optimization of fuel cost while reducing communication and computation burdens by designing a leaderless distributed consensus protocol and combining it with linear parameter variation modeling [13]. I. Ahmed et al. again proposed a consensus-driven based solution for the energy scheduling problem. The incremental cost function was developed through a polynomial curve fitting approach. A partially distributed consensus approach was utilized to handle the incremental cost problem associated with multiple fuel sources, thus effectively addressing the challenge of supply-demand imbalance and facilitating consensus among stakeholders [14]. I. Ahmed et al. also proposed an ET consensus base protocol that considered ramp rate limitation and random false data injection attacks for the distributed power allocation problem in microgrids. The incremental cost information of neighboring generators and local power mismatch information were utilized, thus enhancing the resilience and efficiency of power dispatch under the risk of cyber-attacks and system constraints [15]. I. Ahmed et al. proposed a new consensus oriented distributed approach for the event-triggered ET problem considering ramp rate constraints and integrating green energy sources in a smart grid. The Zeno behavior is eliminated and efficient utilization of communication resources was achieved by transforming the consideration of slope rate constraints into minimum and maximum bounds on the derivatives of generator generation. Meanwhile, this was achieved by developing the Karush-Kuhn-Tucker condition and its approximation to determine the optimality conditions [16].

ALO, as an intelligent optimization algorithm proposed in recent years, has been widely applied and researched in industrial fields such as electrical and power. N. Sridhar and M. Kowsalya proposed combining ALO with the bat algorithm to obtain the optimal energy management strategy for power management. On the foundation of the power grid’s complex requirements, the actual power and reactive power were controlled for power sharing on the generation side. The actual power and reactive power of power management were improved by using a hybrid algorithm to manage the power of the navigation instrument [17]. N. C. Patel et al. proposed a fractional order fuzzy proportional integral derivative controller to ensure the stable operation and control of PS. By utilizing ALO, different controller parameters were optimized. The objective function was integration time’s absolute error. The fractional order fuzzy proportional integral derivative controller had superior robustness under random system loads and was less affected by parameter changes [18]. Y. Bekakra et al. put forward a novel predictive direct power controlling method for parallel active power filters. This reduced the overshoot of DC link voltage changes. Predictive control of filters was achieved by utilizing the Grey Wolf Optimizer and ALO. In the experiment, the power ripple was minimized to the maximum extent in accordance with the standard low battery distortion [19]. G. V. B. Chary and K. M. Rosalina et al. described the impact of minimizing the sum of squared normal deviations on the modeling of transmission lines in ELD to achieve stable and safe operation of all PS. By improving the exploration and utilization characteristics of ALO, the allocation problem of four versions of PS models was solved, balancing load demand and loss [20]. H. Hardiansyah and J. Junaidi proposed a multi-objective ALO metaheuristic algorithm to solve the environmental and economic scheduling problem considering transmission losses. By improving ant-lions and ants’ interaction principle, ants’ fitness value was evaluated using an objective function. The optimal minimum fuel cost and nitrogen oxide emission strategy were ultimately achieved [21]. P. G. Durgut et al. proposed an improved SHuffled Ant-Lion Optimization method for ALO in response to the limitations of ALO in solving complex or large-scale problems in terms of the high number of iterations and the tendency to fall into local optimums. This method improved the accuracy of ALO in solving unconstrained and constrained problems [22].

In summary, relevant scholars have conducted various studies on the ELD and EELD solutions of PS. However, most studies only consider the cost of thermal power generation, ignoring the power loss dissipated in the form of thermal energy during the electricity transmission. Existing literature has made various improvements and optimizations to ALO. However, the accuracy requirements of ELD and EELD for intelligent optimization algorithms still cannot be met. Therefore, this study innovatively utilizes CGO to improve ALO, optimizes ant movement step size using chaotic adjustment factors, as well as modifies and optimizes the operational stage of ALO. On this basis, using IALO to solve ELD and EELD can improve the power quality of PS and enhance load dispatch performance. Finally, the study further compares the differences between the existing load allocation methods and the proposed method, as shown in Table 1.

**Table 1:** Comparison of different methods.

Type	Objective	Constraint	Approach	Reference	Published year
Thermal	Cost model	Valve point effect, prohibited operation area, transmission loss, and other factors	New improved arithmetic optimization algorithm based on elementary function disturbance	W. K. Hao et al. [7]	2022
Thermal	Cost model	Ramp rate limits, prohibited operating zones	Memetic sine cosine algorithm with memory technique	M. A. Al-Betar et al. [8]	2023
Thermal	Cost model	Valve point loading effect	Hybrid of cuckoo search algorithm and Jaya algorithm	S. Basak et al. [9]	2022
Thermal	Cost model; Emission model	Power generation limit, power demand, and power losses evaluation	Multi-objective adaptive real quantum inspired evolutionary algorithm	J. Singh et al. [10]	2023
Thermal; Wind	Cost model; Emission model	Emission limitations, fuel supply constraints, and grid demand response	Equilibrium optimizer for multi-objective dynamic economic emission dispatch model including renewable energy and plug-in electric vehicles	J. Soni and K. Bhattacharjee [11]	2024
Thermal	Cost model	Line losses, generation capacity, valve-point loading effect, prohibited zones, and multi-fueling option on standard IEEE bus systems	Genetic algorithm is used as the initial optimizer, and sequential quadratic programming is employed to fine tune the pre-optimized operation of the genetic algorithm	I. Ahmed et al. [23]	2022
Thermal	Cost model	Electric power research institute, stochastic, peak, and off-peak charging	Search and rescue algorithm; adaptive neuro-fuzzy interference system	I. Ahmed et al. [24]	2023
Thermal	Cost model; Emission model	Considering valve point effects, power balance, generator operating limits, and output constraints	ALO; CGO	This study	-

## 3. Methods and materials

The study first introduces the theoretical knowledge of ALO to improve the performance of PS load allocation. Secondly, ALO is improved and optimized on the basis of CGO. Finally, based on IALO, the design of solution methods for the ELD problem and EELD problem is carried out, respectively.

### 3.1 The ALO algorithm

**Fig 1:**
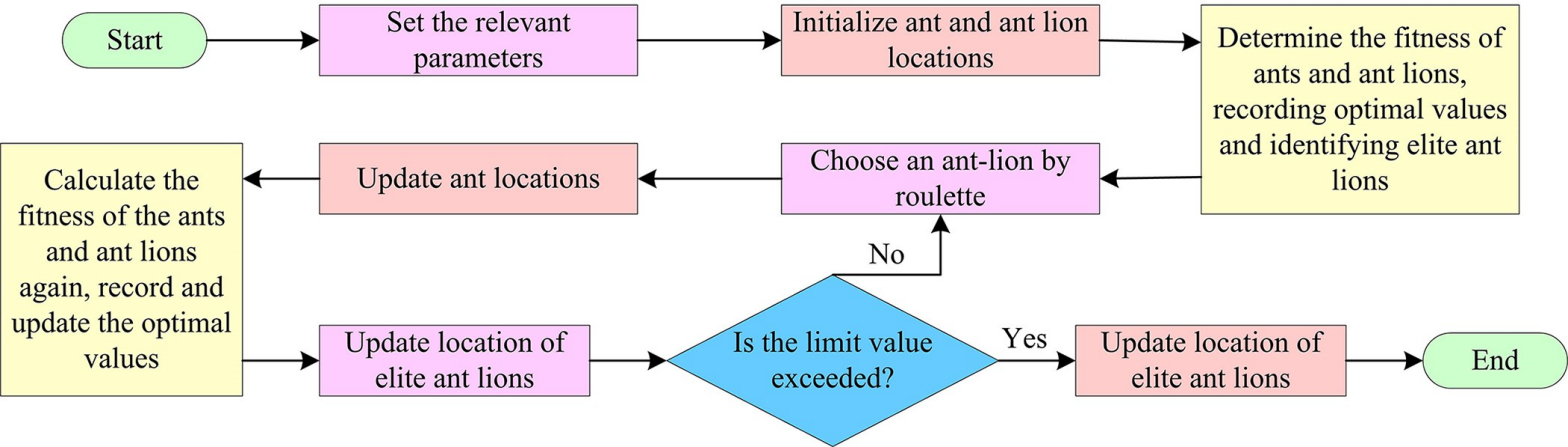
The ALO algorithm optimization flowchart.

ALO solves the problem by mimicking the predatory mechanism of ant-lions in nature. ALO mainly includes five main steps: ant random walking, constructing traps, ant trapping in traps, capturing prey, and rebuilding traps [25]. Fig 1 shows the specific calculation.

In Fig 1, when ants randomly walk in the search space of ant-lions searching for food, their step set is represented by Eq (1).


$$\{\begin{array}{l} X(t)=[0,C_{sum}(2r(t_{1})-1),\ldots ,C_{sum}(2r(t_{n})-1)]\\ r(t)=\{\begin{array}{cc} 1, & rand>0.5\\ 0, & rand\leq 0.5 \end{array} \end{array}$$ (1)

In Eq (1), *X*(*t*) refers to the set of steps taken by ants. *C*_*sum*_ refers to calculating the cumulative total. *t* refers to the current iteration. *n* refers to the maximum iteration. *r*(*t*) refers to a random function. *rand* represents a uniformly distributed random number. Normalization is commonly utilized to ensure that ants always roam within the ant-lion’s searchable space, so the position update of ants is represented by Eq (2).


$$X_{i}^{t}=\frac{\left(X_{i}^{t}-\alpha_{i})(p_{i}^{t}-q_{i}^{t}\right)}{\left(\beta_{i}-\alpha_{i}\right)}+q_{i}^{t}$$ (2)

In Eq (2), $X_{i}^{t}$ refers to ant *i*’s position at the *t*th iteration. *α*_*i*_ and *β*_*i*_ correspond to the minimum and maximum number of steps for ants to randomly walk. $p_{i}^{t}$ and $q_{i}^{t}$ correspond to the minimum and maximum values generated by ants during iteration. Therefore, the positions and fitness of all ants are represented by Eq (3).


$$M_{A}=[\begin{array}{cccc} A_{1,1} & A_{1,2} & \cdots & A_{1,j}\\ A_{2,1} & A_{2,2} & \cdots & A_{2,j}\\ \vdots & \vdots & \ddots & \vdots \\ A_{i,1} & A_{i,2} & \cdots & A_{i,j} \end{array}],\;M_{Af}=[\begin{array}{c} f([A_{1,1},A_{1,2},\cdots ,A_{1,j}])\\ f([A_{2,1},A_{2,2},\cdots ,A_{2,j}])\\ \vdots \\ f([A_{i,1},A_{i,2},\cdots ,A_{i,j}]) \end{array}]$$ (3)

In Eq (3), *M*_*A*_ refers to the position of all ants. *A*_*i*,*j*_ refers to the *i*th ant’s value in the *j*th variable. *M*_*Af*_ refers to the fitness of all ants. *f* refers to the fitness function. According to the principle of updating the position of ants, the position and fitness of ant-lions can be further obtained, represented by Eq (4).


$$M_{L}=[\begin{array}{cccc} L_{1,1} & L_{1,2} & \cdots & L_{1,j}\\ L_{2,1} & L_{2,2} & \cdots & L_{2,j}\\ \vdots & \vdots & \ddots & \vdots \\ L_{i,1} & L_{i,2} & \cdots & L_{i,j} \end{array}],\;M_{Lf}=[\begin{array}{c} f([L_{1,1},L_{1,2},\cdots ,L_{1,j}])\\ f([L_{2,1},L_{2,2},\cdots ,L_{2,j}])\\ \vdots \\ f([L_{i,1},L_{i,2},\cdots ,L_{i,j}]) \end{array}]$$ (4)

In Eq (4), *M*_*L*_ and *M*_*Lf*_ correspond to the positions and fitness of all ant-lions. *L*_*i*,*j*_ represents the *i*th ant-lion’s value located in the *j*th variable. When ants randomly walk within the searchable space of ant-lions, the young ant-lions construct traps. The constructed trapping ability is directly proportional to the ant-lion’s fitness. Therefore, during the optimization, ALO will first use roulette wheel for ant-lion selection [26], represented by Eq (5).


$$\{\begin{array}{c} p_{i}^{t}=L_{j}^{t}+p^{t}\\ q_{i}^{t}=L_{j}^{t}+q^{t} \end{array}$$ (5)

In Eq (5), *p*^*t*^ and *q*^*t*^ correspond to the minimum and maximum values of all variables during the iteration. $L_{j}^{t}$ represents the ant-lion’s position. When an individual realizes that an ant has entered a trap, it will spray sand outward from the center of the trap to prevent the ant from escaping [27]. At this point, the spatial range of ant random walks adaptively decreases, represented by Eq (6).


$$\{\begin{array}{c} p^{t}=\frac{p^{t}}{I}\\ q^{t}=\frac{q^{t}}{I}\\ I=\frac{t}{T}10^{\omega } \end{array}$$ (6)

In Eq (6), *I* refers to the ratio of the current iteration to the maximum iteration. *T* refers to all iterations. *ω* refers to a constant defined based on the current iteration. When ants reach the center of the trap, they will be captured by the ant-lion. At this point, the adaptability of ants is higher than that of ant-lions. The location of the successfully captured ant-lion will be updated to the location of the hunted ant. This process is represented by Eq (7).


$$if\;f(X_{i}^{t})<f(L_{j}^{t}),\;L_{j}^{t}=X_{i}^{t}$$ (7)

### 3.2 Improvement strategy for IALO based on CGO

In traditional ALO, the best ant-lion obtained in each iteration is retained as the elite ant-lion. Only elite ant-lions can have an impact on the random walks of all ants throughout the entire optimization process. Assuming that each ant randomly walks between the ant-lion and the elite ant-lion, the roulette wheel will be more inclined to choose the elite ant-lion. This can lead to poor search performance in the algorithm. At this time, the position equation of the *i*th ant in the *t*th generation is shown in Eq (8).


$$X_{i}^{t}=\frac{(R_{X}^{t}=R_{L}^{t})+R_{L}^{t}}{2}=R_{L}^{t}$$ (8)

In Eq (8), $R_{X}^{t}$ refers to the ant positions that optionally walk around an ant-lion when iterating. $R_{L}^{t}$ refers to the ant positions that optionally walk around the elite ant-lion when iterating. According to Eq (8), the wandering of most ants is limited by the elite ants. This is rather a disadvantage of ALO facing poor survey performance. Therefore, this study modifies the elite stage of ALO by adding an elite weight to control the probability of ants randomly wandering around the elite ant-lion. The modified ant position is represented by Eq (9).


$$X_{iw}^{t}=\frac{(2-w)R_{X}^{t}+wR_{L}^{t}}{2}$$ (9)

In Eq (9), $X_{iw}^{t}$ refers to the modified ant position. *w* refers to the weight of the elite. When its value is 0, ants only walk around the ant colony. When its value is 1, it means the unimproved ALO condition. When its value is 2, ants only randomly walk around the elite ant-lion. When solving ALO, random search cannot traverse the space. Therefore, the study introduces CGO to improve the random search of ALO. CGO is an optimization method based on chaos theory, which enhances the search capability of the algorithm by introducing chaotic dynamics. In the CGO algorithm, the characteristics of chaos are transformed into disordered forms by breaking the rules of the original ordered motion forms. This further leads to chaotic search in traversal space, achieving higher computational efficiency [28]. Therefore, the study utilizes logistic mapping to incorporate the chaotic variables of CGO into the movement step size of ants, represented by Eq (10).


$$\{\begin{array}{l} x_{n+1}=ux_{n}(1-x_{n})\\ X_{i}^{t}=x_{n+1}\frac{\left(X_{i}^{t}-\alpha_{i})(p_{i}^{t}-q_{i}^{t}\right)}{\left(\beta_{i}-\alpha_{i}\right)}+q_{i}^{t} \end{array}$$ (10)

**Fig 2:**
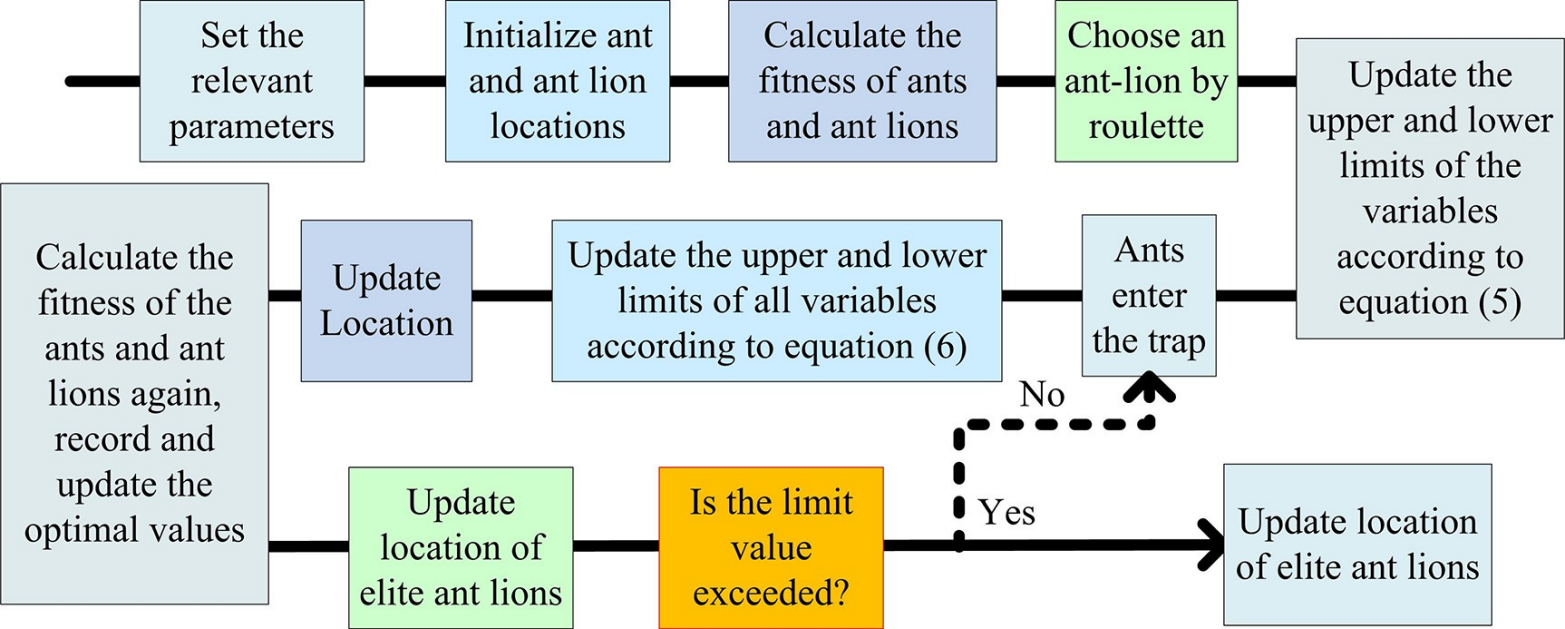
The IALO algorithm solution flowchart.

In Eq (10), *x*_*n*+1_ represents the chaotic adjustment factor. *x*_*n*_ represents the state of the logistic map. When the value is in the range of (0,1), it is in a chaotic state. *u* represents the Logistic parameter. In IALO, the study sets the Logistic parameter to 4. Therefore, Fig 2 shows the final IALO calculation process.

In Fig 2, first, IALO sets the relevant parameters, maximum iteration, total ants and ant-lions, and the range of variable values. After initializing ants and ant-lions’ positions, their fitness is calculated separately. Using roulette wheel to select ant-lions, ants randomly walk within the selected ant-lion search space and update the upper and lower limits of variables according to Eq (5). When the ant enters the trap, all variables’ upper and lower limits are updated using Eq (6), and the position is updated according to Eq (9). The position of the ants is updated according to Eq (8). The ants’ fitness values are calculated. The elite ant-lion’s optimal fitness and the position are recorded and updated. The ant-lion’s new trap is adjusted and optimized according to Eq (7). If the current iteration exceeds the limit value, the output is optimal. Otherwise, the ant-lion selection is repeated.

### 3.3 ELD solving method based on IALO

On the foundation of the improved IALO, a PSELD solution method is designed. This takes into account the cost of thermal power generation and the power loss dissipated in the form of thermal energy during energy transmission. In the entire thermal power generation, there is the consumption characteristic between the energy consumed by the generator unit’s per unit time and the active power output by this unit. When the intake valve of the steam turbine starts, a "wire pulling phenomenon" will occur. The VPE is formed by the pulsating effect attached to the consumption curve [29]. The entire process is represented by Eq (11).


$$\{\begin{array}{l} F_{i}(P_{i})=a_{i}P_{i}^{2}+b_{i}P_{i}+c_{i}\\ E_{i}(P_{i})=|g_{i}\;\sin[h_{i}(P_{i}-P_{i\min})]| \end{array}$$ (11)

In Eq (11), *F*_*i*_(*P*_*i*_) refers to the consumption characteristics of the unit. *P*_*i*_ refers to the active power output by the power generation unit. *a*_*i*_, *b*_*i*_, and *c*_*i*_ refer to the power generation unit’s energy consumption parameters. *E*_*i*_(*P*_*i*_) refers to VPE. *g*_*i*_ and *h*_*i*_ both refer to the VPE characteristic constant terms of the generator set. *P*_*i*min_ means the lowest active power output by the power generation unit. On this basis, the total fuel cost’s objective function of ELD is represented by Eq (12).


$$\{\begin{array}{l} FC_{a}=\min\sum_{i=1}^{k}\left(a_{i}P_{i}^{2}+b_{i}P_{i}+c_{i}\right)\\ FC_{b}=\min\sum_{i=1}^{k}\left\{a_{i}P_{i}^{2}+b_{i}P_{i}+c_{i}+|g_{i}\;\sin[h_{i}(P_{i}-P_{i\min})]|\right\} \end{array}$$ (12)

In Eq (12), *FC*_*a*_ refers to the total fuel cost without considering VPE. *FC*_*b*_ refers to the total fuel cost considering VPE. *k* refers to the total thermal power generation unit. According to the proposed objective function mathematical model, the study constrains the ELD from three aspects: power balance, generator operating limit, and output [30]. Eq (13) is a specific expression.


$$\{\begin{array}{l} \sum_{i=1}^{k}P_{i}=P_{S}+P_{V}\\ P_{S}(t)=\sum_{i=1}^{k}\sum_{j=1}^{k}P_{i}(t)B_{ij}P_{j}(t)+\sum_{i=1}^{k}B_{0i}P_{i}(t)+B_{00}\\ P_{i\min}\leq P_{i}\leq P_{i\max},i=1,2,\ldots ,k \end{array}$$ (13)

In Eq (13), *P*_*S*_ represents the total loss on the transmission line. *P*_*V*_ refers to the total load demand. *B*_*ij*_, *B*_0*i*_, and *B*_00_ refer to the network loss coefficient matrix in the B-coefficient method. *P*_*i*max_ refers to the highest value of active power output by the power generation unit. The output constraint of the generator set is mainly achieved by upper and lower limits. When the output of the assigned unit is less than the lower limit, the output value is adjusted to the lower limit value. When the output of the unit exceeds the upper limit, the output is adjusted to the upper limit value [31]. In ELD, the objective function is the dependent variable, which corresponds to the ant-lion fitness value in IALO. The output power of each unit is an independent variable, corresponding to the ant-lion position in IALO.

It is worth mentioning that ELD refers to Economic Load Dispatch, which is the process of distributing the required load among available generating units to minimize operating costs. ED is a concept in the operation of power systems, involving the allocation of electricity generation to various generating units at the lowest cost while meeting system demand and safe operation. The research mainly addresses the ELD problem, focusing on minimizing the total fuel cost or operating cost by reasonably allocating the output of each generating unit under the premise of power system power balance. Unlike ED, which focuses more on the economic operation of the power system, including but not limited to generation costs, it may also include transmission costs, distribution costs, and costs related to the operation of the electricity market.

### 3.4 EELD solving method based on IALO

EELD not only needs to consider economic benefits, but also needs to consider environmental goals. Therefore, the study utilizes the Price Penalty Factor (PPF) to transform EELD from a multi-objective optimization problem to a single one, and then solves it using IALO. EELD needs to meet the two constraints of power balance and generator operation limits and set the same environmental cost weight and thermal power generation cost weight. Its objective function is represented by Eq (14) [32].


$$\{\begin{array}{l} TC=\min\{FC_{a}(orFC_{b})+PPF\cdot ET\}\\ ET=\sum_{i=1}^{k}(10^{-2}\times (\lambda_{i}+\mu_{i}P_{i}+\vartheta_{i}P_{i}^{2})+\varphi_{i}\exp(\varepsilon_{i}P_{i})\\ PPF[i]=\frac{[FC(P_{i\max})+ET(P_{i\max})]/P_{i\max}}{ET(P_{i\max})/P_{i\max}} \end{array}$$ (14)

**Fig 3:**
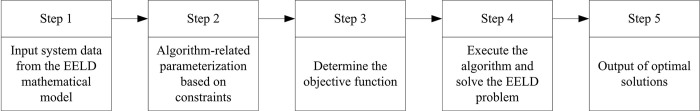
EELD problem solving process based on IALO algorithm.

In Eq (14), *TC* refers to the total objective function of EELD. *FC*_*a*_(*orFC*_*b*_) refers to the economic target in EELD being *FC*_*a*_ or *FC*_*b*_. *PPF* refers to the price penalty factor. *ET* refers to the total emissions of PS. *λ*_*i*_, *μ*_*i*_, *ϑ*_*i*_, *φ*_*i*_, and *ε*_*i*_ refer to the pollution emission indices of power generation units. EELD calculates the maximum output power generation cost based on the equation for the entire process of the thermal power generator. EELD evaluates the average cost of each unit at maximum output. Secondly, the amount of pollution gas emissions generated by each generator at maximum output is determined based on the total emission level. The PPF is obtained based on the average power generation cost and the average pollution gas emissions. Fig 3 shows the process of using IALO to solve EELD.

In Fig 3, firstly, the system parameters of EELD are inputted. The relevant parameters and objective function are determined based on the constraint conditions. Secondly, the objective function is solved using IALO. The optimal load dispatch method, optimal values for power generation cost, pollutant emissions, and the overall objective function are output.

## 4. Results

The study firstly carried out the performance validation of IALO in the application of PS ELD problems and EELD problems. Secondly, experimental simulation validation for solving the ELD problem was carried out. Finally, the performance of IALO was validated by simulation in the EELD problem.

### 4.1 The IALO algorithm performance validation

The study compared the performance of IALO with ALO and the Particle Swarm Optimization (PSO) algorithm to verify the superiority of IALO. Two standard test functions were selected for the validation experiments, mainly including the Sphere function with multidimensional single peaks and the Griewank function with complex multidimensional multiple peaks [33]. Meanwhile, the study set the maximum iteration of the experiments to 600. The number of basic populations was all 30. The results of running 30 experiments for each test function were counted. The maximum number of iterations was set to 600 to ensure that the algorithm had enough time to explore the solution space and strike a balance between convergence and computational efficiency. The basic population size was set to 30 to ensure the diversity of the algorithm and avoid premature convergence to local optima. The outputs of the three algorithms in the four functions are shown in Table 2.

**Table 2:** Performance validation of different algorithms in two benchmark functions.

Target	Sphere function			Griewank function		
	IALO	ALO	PSO	IALO	ALO	PSO
Optimal value	9.63E-66	3.41E-62	6.75E-16	8.69E-4	9.58E-3	8.91E-1
Worst value	4.82E-64	4.52E-53	7.78E-14	1.74E-2	7.87E-2	1.68
Mean value	9.75E-65	1.69E-58	2.78E-15	4.64E-3	4.54E-2	9.26E-1
Standard deviation	6.78E-46	3.47E-25	3.71E-6	9.61E-17	1.74E-12	3.45E-7
Average iteration	500	600	550	350	365	600
Average running time (s)	2.67	3.52	4.53	1.64	3.68	9.44

From Table 2, the proposed IALO algorithm achieved smaller optimal values in the optimization of the two benchmark functions. Comparing the number of iterations and running time of IALO with ALO and PSO on the Sphere function, IALO reduced the average number of iterations by 13.04% and 33.66%, respectively. On the Griewank function, IALO required only 350 iterations with an average running time of 1.64s. Compared to the other two algorithms, the IALO’s iteration speed was significantly more advantageous. This further proved the superiority of IALO in terms of optimization performance and computational efficiency. At the same time, the time complexity analysis of IALO was further conducted.

In the IALO algorithm, the time complexity of the initialization phase is O(N×M). N is the number of individuals, and M is the problem dimension. During the iteration of IALO, each ant updates its position and recalculates the fitness. The time complexity required for this phase is O(I×N×M). I means the number of iterations. When performing elite selection, weight sorting is involved, and the time complexity is closer to O(N×logN). Since IALO introduces a chaotic search mechanism, its time complexity is O(I×N×M). Combining the above, the final time complexity of IALO can be approximately estimated as O(I×N×M+N×logN). Considering the iteration efficiency, it can be seen that although the time complexity of the IALO algorithm has increased, the IALO algorithm has demonstrated superior convergence speed and efficiency on the Sphere and Griewank functions. This means that despite the higher time complexity, the algorithm can effectively find solutions, especially in multi-dimensional multi-peak optimization problems. As the problem size increases, the computational burden of the IALO algorithm will also increase, which may limit its application in large-scale problems.

### 4.2 Verification of ELD solution based on IALO

Firstly, the study utilized the proposed IALO for ELD solving to validate its effectiveness. Simulation experiments were conducted on the 3-unit and 6-unit testing systems in Matlab r2020a software. The simulation was based on the local node units of a large PS. Firstly, the three unit testing system ELD was divided into two scenarios: considering the power loss (network loss) emitted in the form of thermal energy during the electrical energy transmission process and not considering it. The *a*_*i*_, *b*_*i*_, and *b*_*i*_ of Unit 1 correspond to 0.0355, 38.3055, and 1243.5311. *P*_*i*min_ and *P*_*i*max_ correspond to 35 MW and 210 MW. The *a*_*i*_, *b*_*i*_, and *b*_*i*_ of Unit 2 correspond to 0.0211, 36.3278, and 1658.5696. *P*_*i*min_ and *P*_*i*max_ correspond to 130 MW and 325 MW. The *a*_*i*_, *b*_*i*_, and *b*_*i*_ of Unit 3 correspond to 0.0180, 38.2704, and 1356.6592. *P*_*i*min_ and *P*_*i*max_ correspond to 125 MW and 315 MW. When three units meet different load demands (350 MW, 450 MW, and 500 MW), the optimal ELD output of IALO is shown in Table 3.

**Table 3:** ELD optimal solution for units to satisfy different load demands.

*P*_*V*_	Without considering network loss			Considering network loss		
	350 MW	450 MW	500 MW	350 MW	450 MW	500 MW
*P*_1_ (MW)	70.30	93.94	105.88	64.97	86.47	97.23
*P*_2_ (MW)	156.27	193.81	212.73	155.98	192.10	210.16
*P*_3_ (MW)	129.21	171.862	193.31	129.04	171.43	192.62
Network loss	5.78	9.61	11.91	-	-	-
*FC* (Rs/hr)	18564.48	23112.36	25465.47	18315.57	22683.15	24924.13

**Fig 4:**
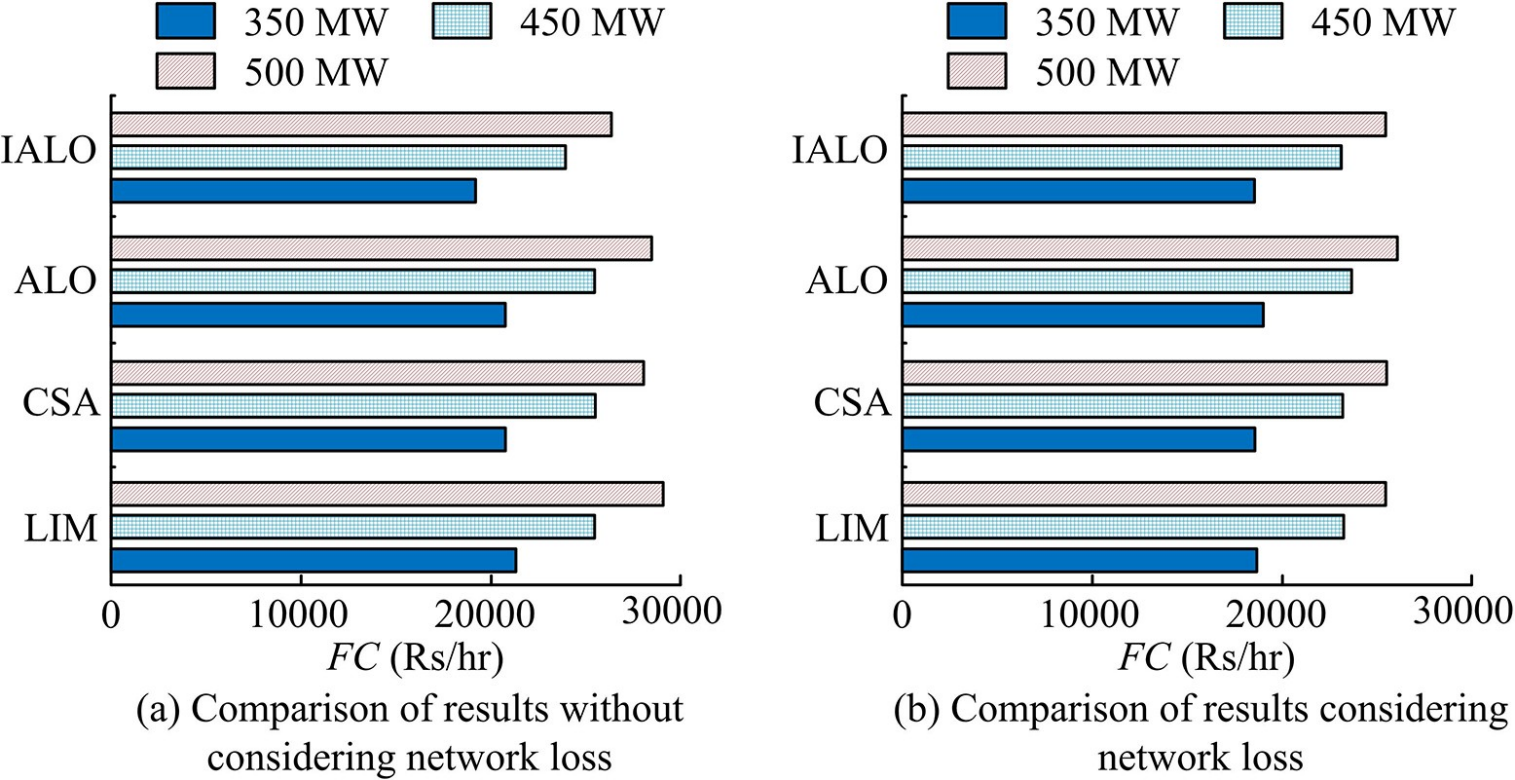
Results comparison of different optimization algorithms for dealing with ELD.

From Table 3, considering network losses, the optimal ELD solutions for the 3-unit outputted by IALO were all within the corresponding extreme values of *P*_*i*min_ and *P*_*i*max_ active power for each unit. The optimal total fuel cost of ELD output without considering network loss was reduced by 1.34%, 1.86%, and 2.13% compared to the total fuel cost considering network loss under different load demands, respectively. This may be because the PS generator unit needs to output more power when considering grid losses, resulting in a higher total fuel cost than the solution without considering grid losses. To further demonstrate the superiority of using IALO to solve ELD, the Lambda Iteration Method (LIM), Cuckoo Search Algorithm (CSA), and the unimproved ALO were compared with the results obtained by IALO. Fig 4 shows the specific comparison results.

In the solution results of Fig 4A without considering network loss, the three load demand results obtained by IALO were significantly better. The essence of solving ELD was to obtain the minimum fuel cost to improve the economic benefits of PS load dispatch. In Fig 4B, considering the solution results of the network, when the load demand was 350 MW, the total fuel cost required for the proposed IALO was reduced by 0.57%, 0.32%, and 2.18% compared to LIM, CAS, and ALO, respectively. When the load demand was 450 MW, the total fuel cost required by IALO was reduced by 0.57%, 0.17%, and 2.39% compared to the three algorithms, respectively. When the load demand was 500 MW, the total fuel cost required by IALO was reduced by 0.18%, 0.10%, and 2.36% compared to the three algorithms, respectively. Under different load demands, the solution results of ALO were significantly worse, indicating that improved ALO was reasonable and reliable.

**Fig 5:**
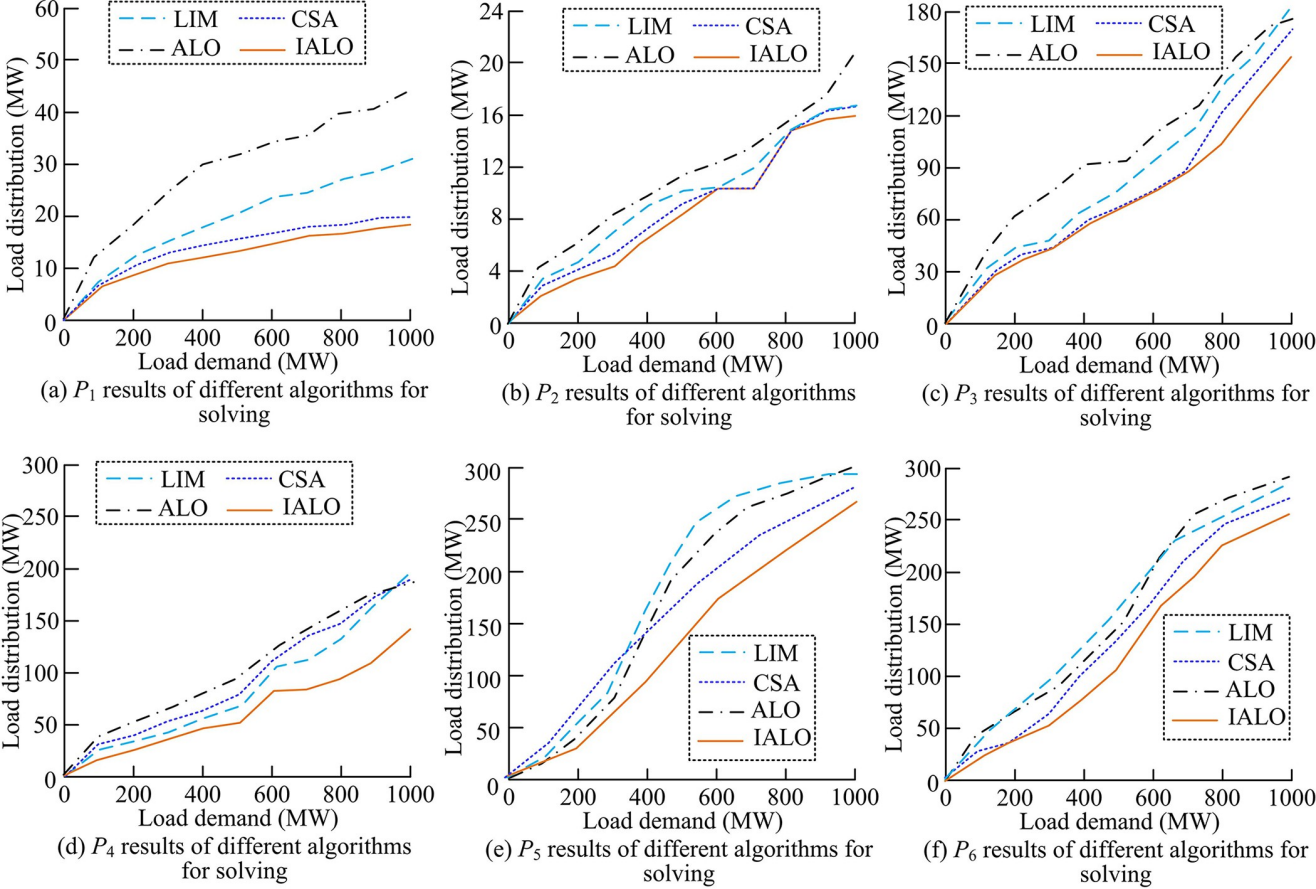
Results of 6-unit load dispatch under different algorithms.

On this basis, further simulation experiments were conducted on the testing system of 6-unit, and ELD solutions were obtained from two scenarios: ignoring VPE and considering VPE. When ignoring VPE for ELD solving, *a*_*i*_, *b*_*i*_, and *c*_*i*_ of Unit 1 correspond to 0.1524, 38.5397, and 756.7989, while *P*_*i*min_ and *P*_*i*max_ correspond to 10 MW and 125 MW. *a*_*i*_, *b*_*i*_, and *c*_*i*_ of Unit 2 correspond to 0.1058, 46.1592, and 451.3251, while *P*_*i*min_ and *P*_*i*max_ correspond to 10 MW and 150 MW. *a*_*i*_, *b*_*i*_, and *c*_*i*_ of Unit 3 correspond to 0.0280, 40.3966, and 1049.9980, while *P*_*i*min_ and *P*_*i*max_ correspond to 35 MW and 225 MW. *a*_*i*_, *b*_*i*_, and *c*_*i*_ of Unit 4 correspond to 0.0354, 38.3055, and 1243.5310, while *P*_*i*min_ and *P*_*i*max_ correspond to 35 MW and 210 MW. *a*_*i*_, *b*_*i*_, and *c*_*i*_ of Unit 5 correspond to 0.0211, 36.3278, and 1658.56, while *P*_*i*min_ and *P*_*i*max_ correspond to 130 MW and 325 MW. *a*_*i*_, *b*_*i*_, and *c*_*i*_ of Unit 6 correspond to 0.0180, 38.2704, and 1356.6590, while *P*_*i*min_ and *P*_*i*max_ correspond to 125 MW and 315 MW. Fig 5 shows the load dispatch results of different algorithms.

**Fig 6:**
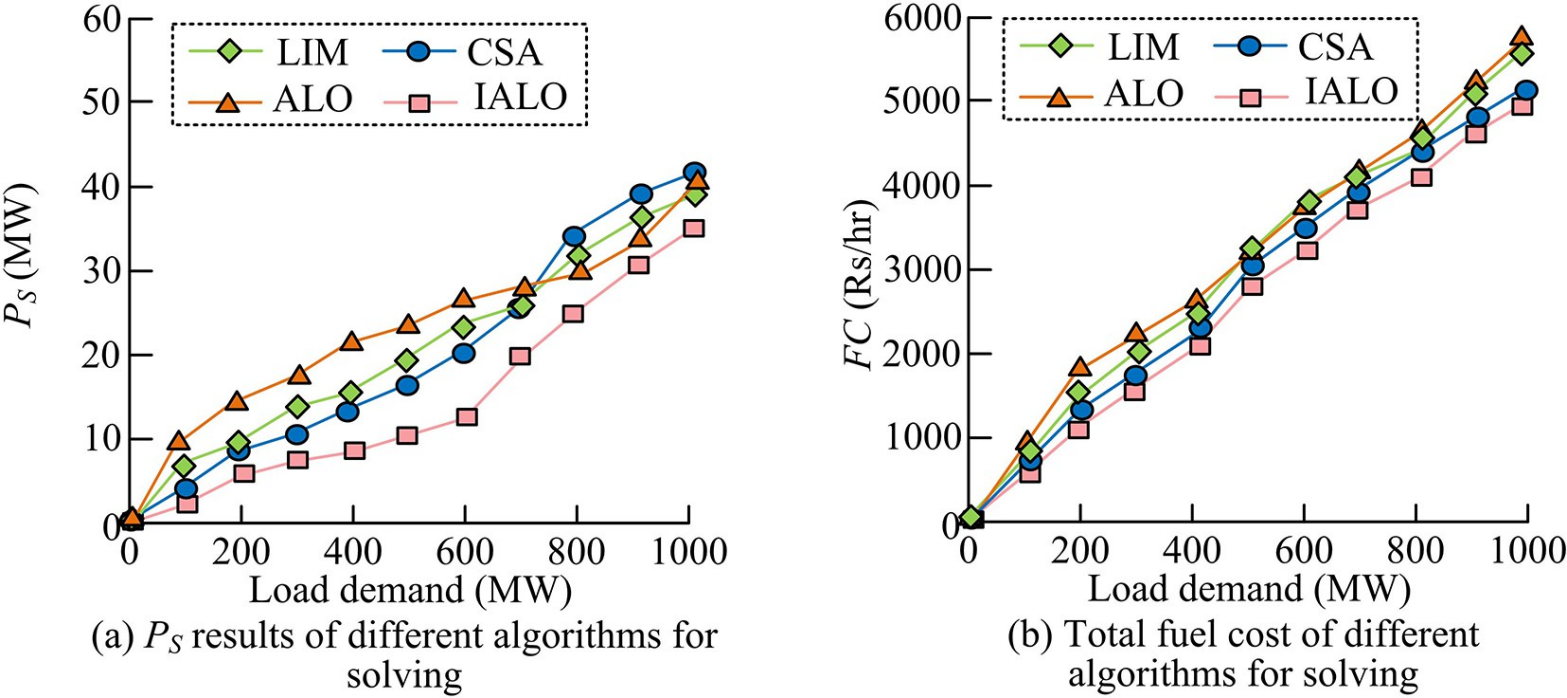
Comparison of optimal results under various load demands.

From Fig 5A–5F, as the load demand increased, the four optimization algorithms showed an upward trend in the calculated results of load dispatch for 6-unit. The overall variation of IALO was smaller than the other three algorithms. The load dispatch result calculated by ALO was significantly the highest. This may be due to the excessive bias of roulette wheel betting towards elite ant-lions during ELD solving, resulting in the algorithm falling into local optima. Although LIM is easy to calculate, its increasing computational complexity makes it difficult to achieve optimal solution to problems. This may be the reason why its solution results are worse than CSA and IALO. Fig 6 shows the optimal total fuel cost and *P*_*S*_ obtained by four algorithms in 6-unit loads.

Fig 6A shows the solution result of the total loss *P*_*S*_ on the transmission line. Low *P*_*S*_ indicates low energy loss and high transmission efficiency in transmission lines. The *P*_*S*_ calculated by IALO under different load demands was the lowest among these four algorithms. IALO solving ELD improved the transmission performance of PS. Compared with the total fuel cost calculated by the four algorithms in Fig 6B, under a load demand of 0–1000 MW, the IALO was reduced by about 6% compared to the results calculated by the other three algorithms. This indicated that in the ELD solution that ignored VPE, IALO obtained superior solution results and improved the efficiency of PS load dispatch.

**Fig 7:**
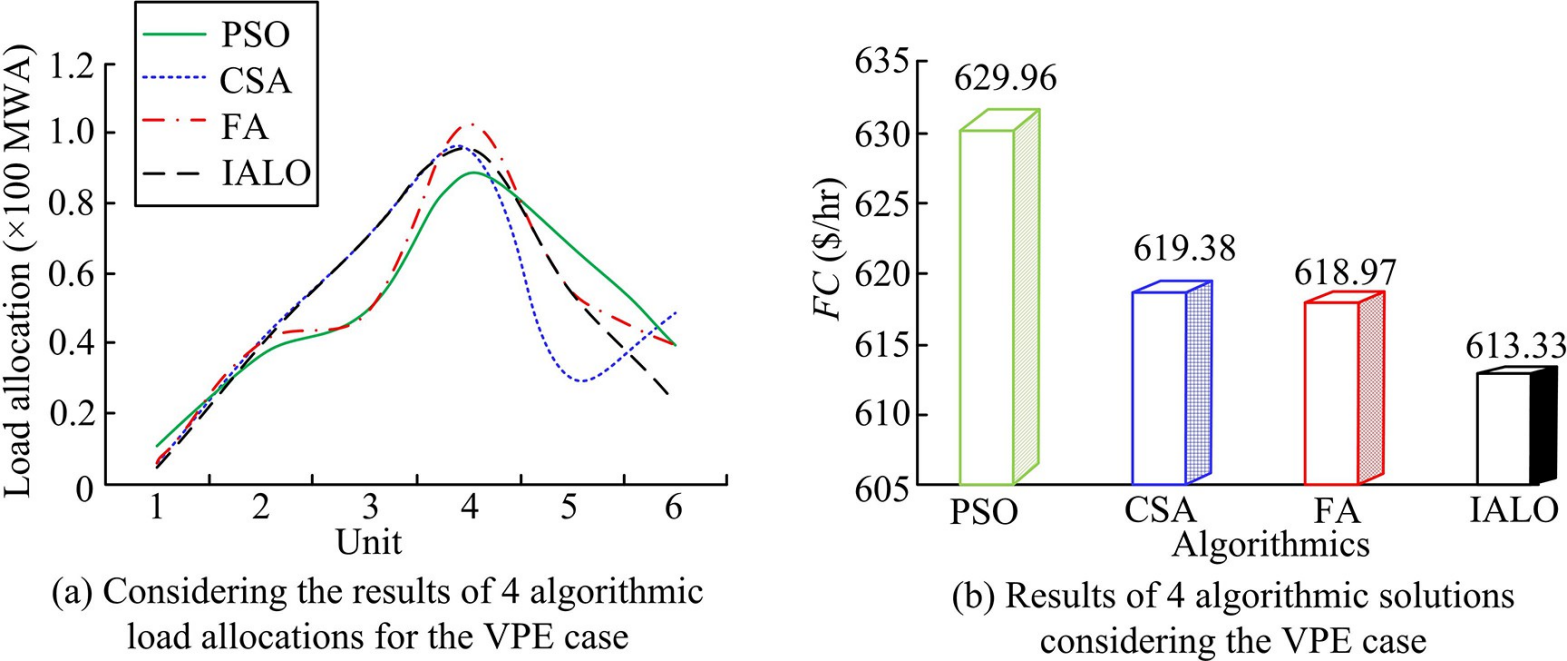
Considering the optimal ELD solution for the VPE case.

Further research was conducted to consider the ELD solution of VPE. The Institute of Electrical and Electronics Engineers (IEEE) 30 nodes with a power demand of 283.40 MW was considered for the 6-unit system. The relevant power parameters were expressed in per unit. *a*_*i*_, *b*_*i*_, *c*_*i*_, *g*_*i*_, and *h*_*i*_ of Unit 1 correspond to 100, 200, 10, 15, and 6.283, while *P*_*i*min_ and *P*_*i*max_ correspond to 0.05 MW and 0.50 MW. *a*_*i*_, *b*_*i*_, *c*_*i*_, *g*_*i*_, and *h*_*i*_ of Unit 2 correspond to 120, 150, 10, 10, and 8.976, while *P*_*i*min_ and *P*_*i*max_ correspond to 0.05 MW and 0.60 MW. *a*_*i*_, *b*_*i*_, *c*_*i*_, *g*_*i*_, and *h*_*i*_ of Unit 3 correspond to 40, 180, 20, 10, and 14.784, while *P*_*i*min_ and *P*_*i*max_ correspond to 0.05 MW and 1.00 MW. *a*_*i*_, *b*_*i*_, *c*_*i*_, *g*_*i*_, and *h*_*i*_ of Unit 4 correspond to 60, 100, 10, 5, and 20.944, while *P*_*i*min_ and *P*_*i*max_ correspond to 0.05 MW and 1.20 MW. *a*_*i*_, *b*_*i*_, *c*_*i*_, *g*_*i*_, and *h*_*i*_ of Unit 5 correspond to 40, 180, 20, 5, and 25.133, while *P*_*i*min_ and *P*_*i*max_ correspond to 0.05 MW and 1.00 MW. *a*_*i*_, *b*_*i*_, *c*_*i*_, *g*_*i*_, and *h*_*i*_ of Unit 6 correspond to 100, 150, 10, 5, and 18.48, while *P*_*i*min_ and *P*_*i*max_ correspond to 0.05 MW and 0.60 MW. The standard voltage of the system is 100 MWA, so the standard value of the load is 2.834 per unit. Based on the above parameter settings, PSO, CSA, Fire-fly Algorithm (FA), and IALO were introduced for solution results and convergence curve comparison in Fig 7.

**Fig 8:**
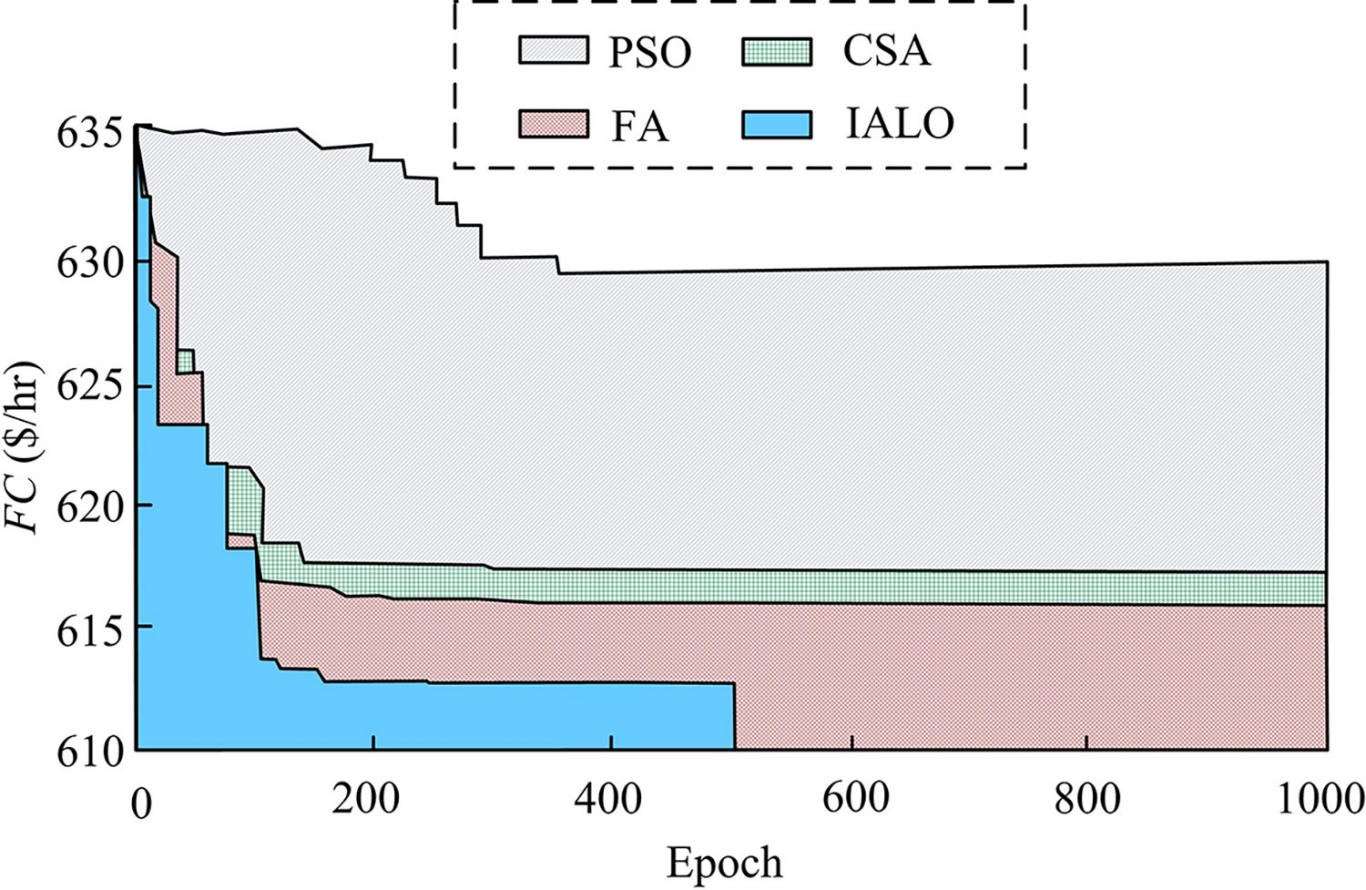
Comparison of convergence curves of different algorithms.

From Fig 7A., the load dispatch results calculated by the four algorithms all conformed to the load per unit value of system 2.834. However, compared to the total fuel cost obtained by the four algorithms in Fig 7B., the cost required for the optimal ELD solution calculated by PSO was 629.96 $/hr, while the proposed IALO was 2.64% less than PSO. Compared to CSA and FA, IALO still had significant advantages. This indicated that IALO still had superiority in solving ELD when considering VPE. The study further analyzed the changes in convergence curves when solving ELD using four algorithms considering VPE in Fig 8.

In Fig 8, IALO had the fastest iteration convergence speed and obtained the optimal value at 500 iterations. The convergence speed of PSO, CSA, and FA algorithms was the slowest. The convergence curve of PSO fluctuated the most, while the iterative convergence curves of the other three algorithms were relatively stable. Overall, the improved IALO significantly improved its optimization performance and improved the accuracy of solving ELD. Based on the above, IALO had significant advantages in solving performance under different unit sizes, demonstrating superior optimization and convergence efficiency in different situations. This indicated that IALO had superior applicability in systems of different scales. Meanwhile, IALO improved its adaptability to PS of different scales by improving the random search mechanism and introducing elite weights.

### 4.3 Verification of EELD solution based on IALO

**Fig 9:**
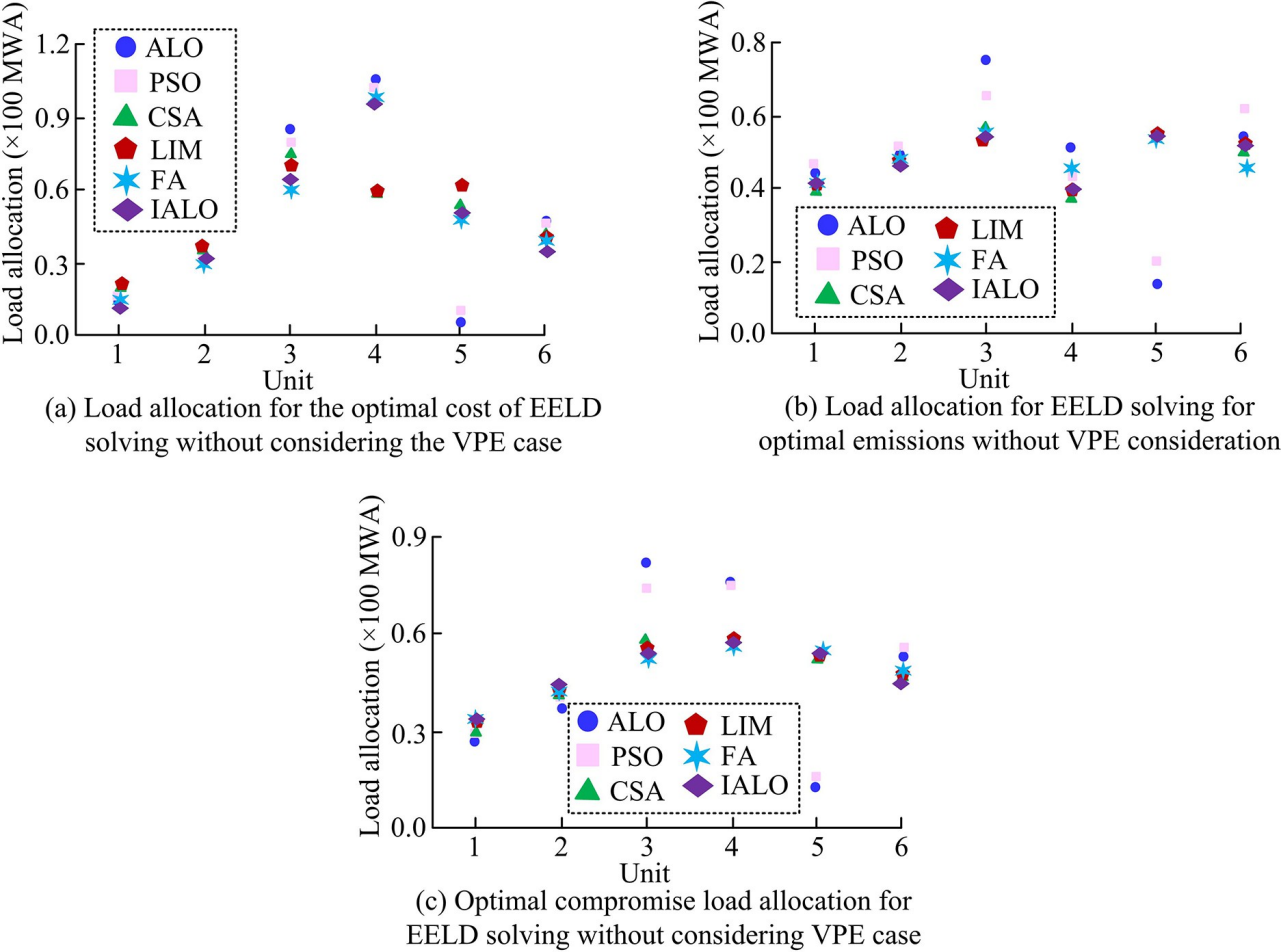
EELD load dispatch results for different algorithms without considering VPE.

IALO has demonstrated superior solving performance in ELD, but its solving efficiency in EELD needs further verification. Therefore, based on ELD, the EELD simulation experiments were conducted. A 6-unit system was set up. The per unit power parameters were consistent with the ELD parameters. In addition, the pollution emission coefficients *λ*_*i*_, *μ*_*i*_, *ϑ*_*i*_, *φ*_*i*_, and *ε*_*i*_ of Unit 1 correspond to 4.091, -5.554, 6.49, 2.0×10^−4^, and 2.857. The pollution emission coefficients *λ*_*i*_, *μ*_*i*_, *ϑ*_*i*_, *φ*_*i*_, and *ε*_*i*_ of Unit 2 correspond to 2.543, -6.047, 5.638, 5.0×10^−4^, and 3.333. The pollution emission coefficients *λ*_*i*_, *μ*_*i*_, *ϑ*_*i*_, *φ*_*i*_, and *ε*_*i*_ of Unit 3 correspond to 4.258, -5.094, 4.586, 1.0×10^−6^, and 8. The pollution emission coefficients *λ*_*i*_, *μ*_*i*_, *ϑ*_*i*_, *φ*_*i*_, and *ε*_*i*_ of Unit 4 correspond to 5.326, -3.55, 3.38, 2.0×10^−3^, and 2. The pollution emission coefficients *λ*_*i*_, *μ*_*i*_, *ϑ*_*i*_, *φ*_*i*_, and *ε*_*i*_ of Unit 5 correspond to 4.258, -5.094, 4.586, 1.0×10^−6^, and 8. The pollution emission coefficients *λ*_*i*_, *μ*_*i*_, *ϑ*_*i*_, *φ*_*i*_, and *ε*_*i*_ of Unit 6 correspond to 6.131, -5.555, 5.151, 1.0×10^−5^, and 6.667. Based on the above parameters, the EELD solution without considering VPE was conducted. The results obtained from IALO were compared ALO, PSO, CSA, LIM, and FA. Fig 9 shows the load dispatch results of six algorithms.

**Fig 10:**
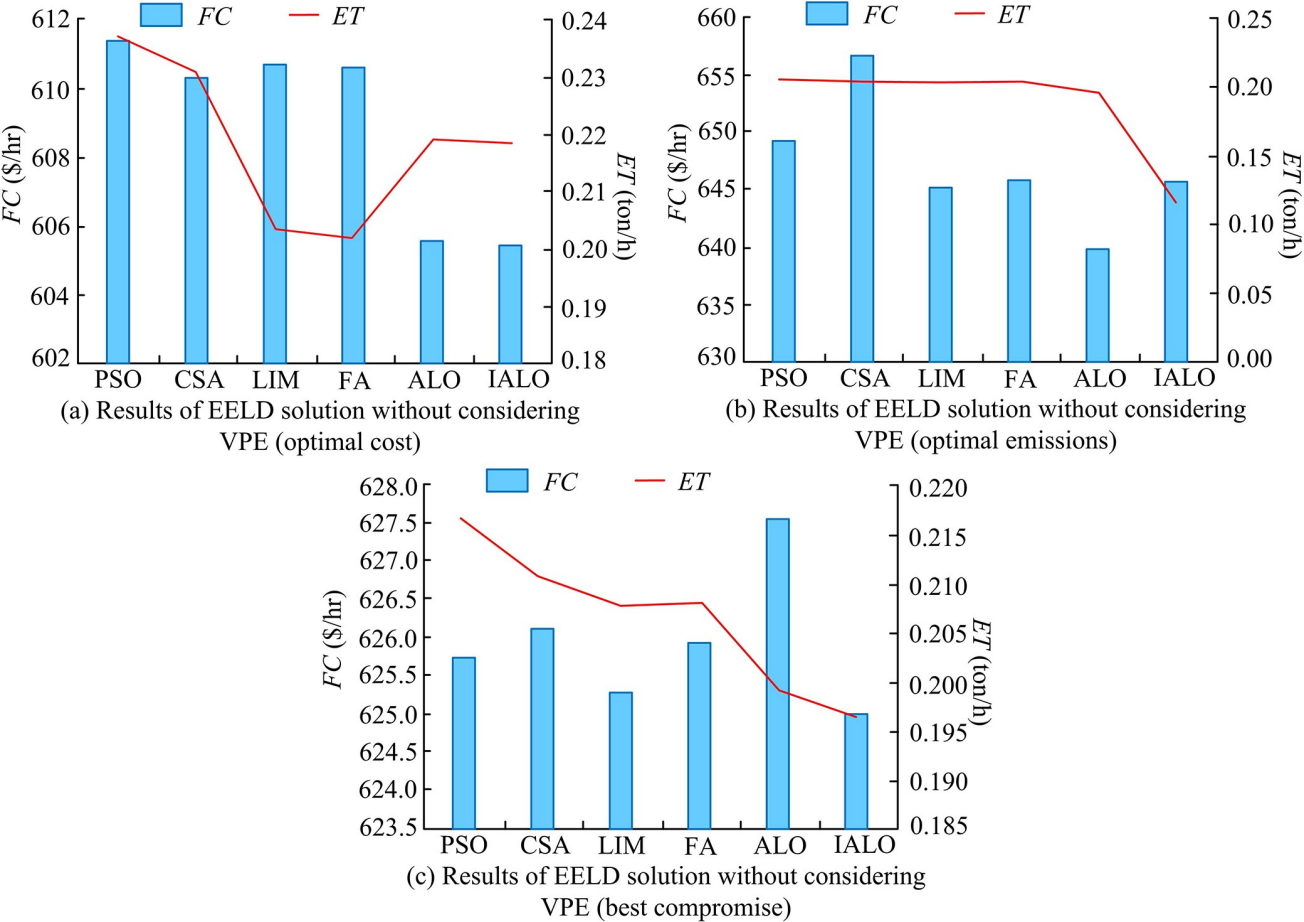
Comparison of results obtained by different algorithms for solving EELD.

Fig 9A shows the optimal cost load dispatch results calculated by six algorithms without considering VPE. In the case of only obtaining the optimal cost, Unit 3 and Unit 4 were allocated more loads. Compared to the optimal emission load dispatch results in Fig 9B, Unit 3 was assigned more loads. Fig 9C shows the load dispatch of the unit while considering both optimal cost and optimal emissions. In a compromise, the load dispatch of Unit 1 and Unit 2 was improved. Fig 10 shows the total fuel cost and emissions of EELD obtained through optimization using six algorithms.

**Fig 11:**
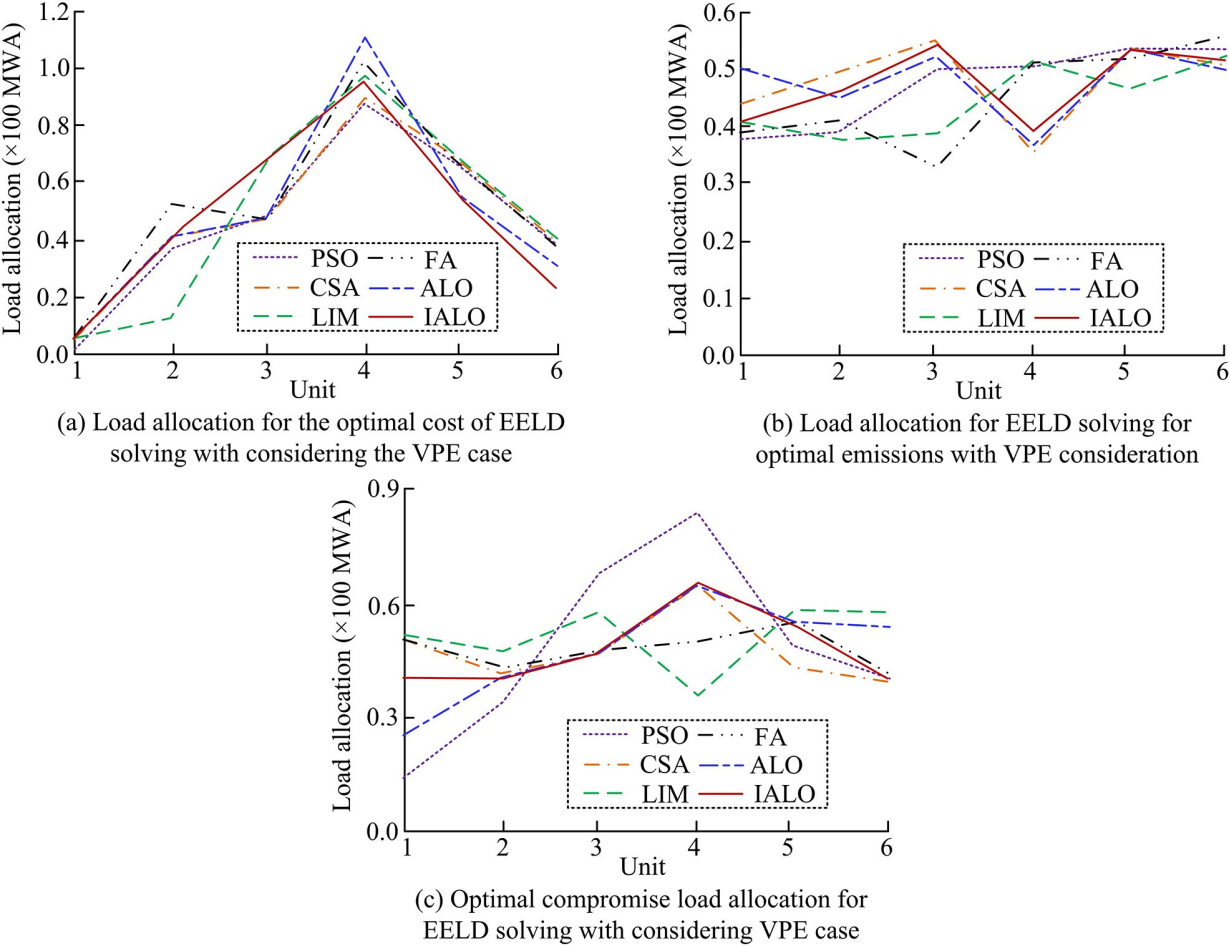
EELD load dispatch results for different algorithms with considering VPE.

After comparing the optimal cost obtained by different algorithms for solving EELD in Fig 10A, the total fuel cost calculated by IALO decreased by an average of 1.39% compared to other algorithms. The total emissions were higher than LIM and FA. This may be because in the process of optimizing based on total fuel cost, the focus of IALO optimization is on economic goals. However, the total emissions of IALO still decreased by an average of 4.41% compared to PSO, CSA, and ALO. For the optimal emissions in Fig 10B, IALO had a clear advantage in solving the environmental objectives of EELD. By comparing the EELD solution obtained by balancing economic and environmental factors in Fig 10C, the optimal total fuel cost obtained by IALO was 625.01 $/hr, and the total emissions were 0.1964 tons/h. Compared with the other 5 algorithms, this scheme reduced the total fuel cost by an average of 1.11 $/hr and the total emissions by 0.012 tons/h. This indicated that IALO had significant advantages in solving EELD without considering VPE. Finally, the study further investigated the EELD solution considering the VPE situation. Fig 11 shows the load of 6-unit calculated by 6 algorithms.

From the optimal cost load dispatch in Fig 11A, with only considering economic objectives, the six algorithms allocated the most load to the 4 units. In Fig 11B, for the optimal emission load dispatch, except for the LIM and FA algorithms, the other four algorithms all allocated more loads to the 3 units. In the solution results of the optimal cost and emissions in Fig 11C, LIM allocated the most load to the 3 units, while FA allocated more load to 5 units. Other algorithms allocated the most load to 4 units. Table 4 shows the total fuel cost and total emissions obtained by solving EELD using six algorithms considering VPE.

**Table 4:** Comparison of results obtained by different algorithms for solving EELD.

Algorithms	Optimal cost		Optimal emissions		Best compromise	
	*FC* ($/hr)	*ET* (ton/h)	*FC* ($/hr)	*ET* (ton/h)	*FC* ($/hr)	*ET* (ton/h)
PSO	626.96	0.25	659.44	0.53	639.65	0.23
CSA	626.28	0.22	648.92	0.52	641.81	0.22
LIM	627.28	0.23	675.88	0.48	639.41	0.21
FA	625.77	0.26	656.98	0.56	651.30	0.22
ALO	619.35	0.23	686.23	0.22	650.45	0.23
IALO	613.32	0.22	674.21	0.19	622.46	0.20

**Fig 12:**
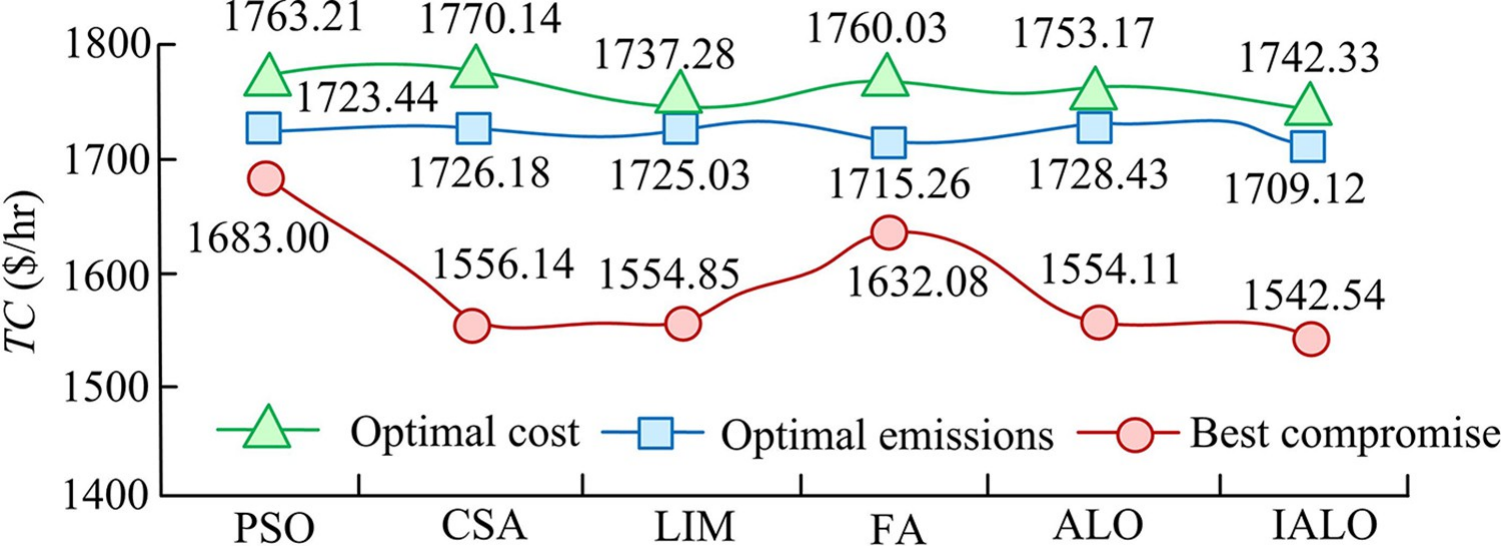
Results comparison of different algorithms for dealing with EELD’s total objective function when VPE is considered.

In Table 4, when solving for EELD as the optimal cost, the total fuel cost of IALO decreased by an average of 11.81 $/hr compared to other algorithms, and the total emissions decreased by an average of 7.56%. Comparing the best emission schemes of six algorithms, the optimal emission of IALO was 0.19 tons/h, which was an average reduction of 0.273 tons/h compared to the other five algorithms. Taking into account the total fuel cost and emissions, the optimal solution calculated by IALO required a total fuel cost of 622.46 $/hr and a total emissions of 0.20 tons/h, which was the most optimal among the six algorithms. Based on the results obtained from the best cost, best emissions, and best compromise, the study further analyzed the total objective function *TC* obtained by solving EELD using six algorithms in Fig 12.

In Fig 12, under the optimal cost solution, the total objective function *TC* calculated by LIM had the lowest result. Under the optimal emission solution, the total objective function *TC* calculated by IALO had the lowest result. Comparing the total objective functions of these six algorithms under the best compromise solution, the *TC* of PSO was 1683.00 $/hr, which was the highest among these six algorithms. This may be due to PSO falling into local optima, resulting in its final *TC* value being too high. The proposed IALO had a *TC* of 1542.54 $/hr, which was an average reduction of 53.55 $/hr compared to the other five algorithms. Therefore, the improved IALO strategy was reasonable and reliable, which was significantly better than other algorithms in solving EELD. IALO showed fast convergence speed on multiple test functions and actual PS models, considering various practical operational constraints including VPE, demonstrating good generality. Compared with other algorithms, IALO showed advantages in multiple evaluation metrics, indicating its good robustness in systems of different scales.

## 5. Conclusion

The study proposes IALO for solving load allocation problems, aiming to improve the efficiency of PS load allocation. The IALO algorithm enhances its global search capability and optimization performance by introducing elite weights and chaotic search mechanism, achieving dual optimization on ELD and EELD problems. Compared with other algorithms, IALO reduced the average number of iterations and running time of Sphere function by 13.04% and 33.66%, respectively. ELD verification showed that IALO under load allocation of 3 units reduced the total fuel cost by 0.10%-2.39% compared to the other 3 algorithms. Under the load distribution of 6 units, IALO required an average reduction of 6% in total fuel cost compared to the 3 algorithms. EELD verification showed that IALO optimized the solution with a total fuel cost of 622.46 $/hr and a total emissions of 0.20 ton/h when considering VPE, which was the most superior among the optimization results of the six algorithms. The overall objective function obtained by IALO optimization was 1542.54 $/hr, which was an average reduction of 53.55 $/hr compared to the other five algorithms. The results indicate that IALO algorithm has superior optimization ability and requires less convergence iteration time. The use of improved IALO algorithm for PS load allocation has positive application value, as it can solve the ELD and EELD problems in practical cases. The IALO algorithm has achieved significant results in improving the efficiency of power system load allocation, reducing operating costs, and environmental benefits. Its research results have important theoretical and practical significance.

However, the study did not take into account the characteristic of prohibiting power in the generator set during the experimental process, which may lead to differences between simulation results and actual situations. In addition, the load allocation considered in the study is all static allocation. Therefore, in the future, machine learning, deep learning, and other methods will be introduced to predict dynamic load allocation, and IALO intelligent algorithms will be used to solve the allocation problem. Consider applying the IALO algorithm to real-time systems to achieve dynamic optimization and control of power system load allocation, and further investigate the performance of IALO algorithm in handling multi-objective optimization problems, especially in complex scenarios that seek balance between economic, environmental, and social objectives. We hope that IALO algorithm can provide more efficient and intelligent optimization solutions for power systems and a wider range of fields.

### 5.1 Findings

IALO has superior convergence speed and efficiency in multidimensional multimodal functions such as Sphere and Griebank functions, which can find more accurate optimal solutions.

IALO can output solutions for ELD and EELD problems with lower fuel costs and emissions in PS systems of different scales, which has positive application significance in PS load allocation.

The final time complexity of IALO can be approximately estimated as O (I×N×M+N×logN).

IALO has ideal applicability and robustness in PS of different scales.

## Supporting information

S1 Dataset (DOC)
