# Supplementary material for: Application of improved ant-lion algorithm for power systems
Source: PLoS One. 2024 Dec 4;19(12):e0311563. doi: 10.1371/journal.pone.0311563 (PMC11616874; doi:10.1371/journal.pone.0311563)
Supplement: S1 Dataset — (DOC) [file pone.0311563.s001.doc]

**The data in Fig. 4**

| Grid size | Consider network loss | | | | Consider network loss | | | |
| --- | --- | --- | --- | --- | --- | --- | --- | --- |
|  | IALO | ALO | CSA | LIM | IALO | ALO | CSA | LIM |
| 350MW | 18564.48 | 18670.90 | 1862477.00 | 19013.19132 | 18315.57 | 18723.75 | 18374.37 | 18420.57 |
| 450MW | 23112.36 | 23678.27 | 23151.72 | 23244.85568 | 22683.15 | 23238.55 | 22721.78 | 22813.19 |
| 500MW | 25465.47 | 19013.19 | 18583.06 | 25511.3905 | 24924.13 | 25526.56 | 24949.08 | 24969.07 |

**The data in Fig. 5 (MW)**

| *P*_1_ | Load demand (MW) | LIM | CSA | ALO | IALO | *P*_2_ | Load demand (MW) | LIM | CSA | ALO | IALO |
| --- | --- | --- | --- | --- | --- | --- | --- | --- | --- | --- | --- |
|  | 200 | 12.4 | 10.7 | 16.3 | 8.2 |  | 200 | 4.1 | 3.8 | 6.7 | 3.1 |
|  | 400 | 16.7 | 14.1 | 29.5 | 11.5 |  | 400 | 7.1 | 6.8 | 10.4 | 6.4 |
|  | 600 | 23.4 | 17.3 | 34.2 | 14.2 |  | 600 | 11.3 | 10.3 | 14.3 | 10.3 |
|  | 800 | 29.8 | 18.1 | 42.4 | 16.3 |  | 800 | 15.4 | 14.8 | 16.7 | 14.6 |
|  | 1000 | 32.4 | 18.9 | 45.2 | 17.4 |  | 1000 | 15.7 | 15.7 | 20.6 | 15.4 |
| *P*_3_ | Load demand (MW) | LIM | CSA | ALO | IALO | *P*_4_ | Load demand (MW) | LIM | CSA | ALO | IALO |
|  | 200 | 44.5 | 37.8 | 57.4 | 31.3 |  | 200 | 30.4 | 37.8 | 55.7 | 18.7 |
|  | 400 | 58.4 | 49.2 | 74.2 | 55.4 |  | 400 | 59.9 | 67.8 | 80.2 | 48.2 |
|  | 600 | 79.0 | 72.4 | 95.3 | 69.2 |  | 600 | 113.4 | 124.5 | 120.4 | 79.2 |
|  | 800 | 150.4 | 139.2 | 150.4 | 110.3 |  | 800 | 148.9 | 153.4 | 150.0 | 109.4 |
|  | 1000 | 179.2 | 167.3 | 171.3 | 157.2 |  | 1000 | 183.4 | 180.3 | 175.2 | 139.8 |
| *P*_5_ | Load demand (MW) | LIM | CSA | ALO | IALO | *P*_6_ | Load demand (MW) | LIM | CSA | ALO | IALO |
|  | 200 | 52.3 | 65.8 | 30.4 | 25.3 |  | 200 | 78.3 | 30.2 | 76.4 | 29.1 |
|  | 400 | 152.3 | 143.4 | 143.4 | 75.5 |  | 400 | 119.3 | 78.4 | 121.4 | 74.5 |
|  | 600 | 262.3 | 213.4 | 234.5 | 167.8 |  | 600 | 187.4 | 169.8 | 225.4 | 160.2 |
|  | 800 | 276.2 | 235.6 | 268.4 | 230.0 |  | 800 | 225.3 | 245.3 | 263.2 | 219.4 |
|  | 1000 | 289.9 | 276.3 | 298.9 | 267.8 |  | 1000 | 270.4 | 267.4 | 278.3 | 239.5 |

**The data in Fig. 6**

| *P_S_* (MW) | Load demand (MW) | LIM | CSA | ALO | IALO | *FC* (Rs/hr) | Load demand (MW) | LIM | CSA | ALO | IALO |
| --- | --- | --- | --- | --- | --- | --- | --- | --- | --- | --- | --- |
|  | 200 | 9.8 | 9.6 | 15.3 | 6.4 |  | 200 | 1674.5 | 1623.5 | 1802.4 | 1200.4 |
|  | 400 | 15.7 | 13.2 | 23.4 | 8.7 |  | 400 | 2342.4 | 2203.4 | 2503.4 | 1872.5 |
|  | 600 | 24.2 | 24.2 | 26.3 | 12.5 |  | 600 | 3781.4 | 3701.9 | 3513.9 | 3245.2 |
|  | 800 | 34.2 | 35.1 | 30.6 | 26.4 |  | 800 | 4422.3 | 4320.4 | 4498.2 | 3892.4 |
|  | 1000 | 39.4 | 43.2 | 41.3 | 34.3 |  | 1000 | 5769.2 | 5231.4 | 5924.1 | 4982.4 |

**The data in Fig. 7**

| Method | Load allocation (×100 MWA) | | | | | | *FC* ($/hr) |
| --- | --- | --- | --- | --- | --- | --- | --- |
|  | 1 | 2 | 3 | 4 | 5 | 6 |  |
| PSO | 0.15 | 0.37 | 0.46 | 0.83 | 0.64 | 0.42 | 629.96 |
| CSA | 0.08 | 0.43 | 0.63 | 0.88 | 0.32 | 0.46 | 619.38 |
| FA | 0.08 | 0.41 | 0.46 | 1.00 | 0.53 | 0.42 | 618.97 |
| IALO | 0.08 | 0.43 | 0.63 | 0.88 | 0.53 | 0.34 | 613.33 |

**The data in Fig. 8 ($/hr)**

| Epoch | PSO | CSA | FA | IALO |
| --- | --- | --- | --- | --- |
| 0 | 635.0 | 635.0 | 635.0 | 635.0 |
| 200 | 633.8 | 617.6 | 616.8 | 612.5 |
| 400 | 628.9 | 617.4 | 616.2 | 612.5 |
| 600 | 628.9 | 617.4 | 616.2 | 612.5 |
| 800 | 628.9 | 617.4 | 616.2 | 612.5 |
| 1000 | 628.9 | 617.4 | 616.2 | 612.5 |

**The data in Fig. 9**

| Unit | ALO | | | PSO | | | CSA | | |
| --- | --- | --- | --- | --- | --- | --- | --- | --- | --- |
|  | (a) | (b) | (c) | (a) | (b) | (c) | (a) | (b) | (c) |
| 1 | 0.26 | 0.45 | 0.28 | 0.15 | 0.46 | 0.31 | 0.27 | 0.40 | 0.31 |
| 2 | 0.42 | 0.48 | 0.38 | 0.40 | 0.50 | 0.40 | 0.41 | 0.47 | 0.40 |
| 3 | 0.87 | 0.74 | 0.76 | 0.85 | 0.67 | 0.74 | 0.84 | 0.52 | 0.59 |
| 4 | 1.12 | 0.52 | 0.72 | 1.10 | 0.45 | 0.72 | 1.11 | 0.36 | 0.58 |
| 5 | 0.08 | 0.18 | 0.14 | 0.10 | 0.23 | 0.20 | 0.57 | 0.53 | 0.56 |
| 6 | 0.46 | 0.54 | 0.52 | 0.46 | 0.67 | 0.55 | 0.44 | 0.48 | 0.52 |
| Unit | LIM | | | FA | | | IALO | | |
|  | (a) | (b) | (c) | (a) | (b) | (c) | (a) | (b) | (c) |
| 1 | 0.28 | 0.41 | 0.33 | 0.17 | 0.42 | 0.33 | 0.14 | 0.40 | 0.33 |
| 2 | 0.42 | 0.46 | 0.40 | 0.31 | 0.48 | 0.39 | 0.34 | 0.46 | 0.40 |
| 3 | 0.83 | 0.52 | 0.57 | 0.61 | 0.54 | 0.55 | 0.63 | 0.53 | 0.56 |
| 4 | 0.60 | 0.37 | 0.58 | 1.09 | 0.46 | 0.56 | 1.07 | 0.47 | 0.58 |
| 5 | 0.64 | 0.53 | 0.56 | 0.54 | 0.53 | 0.55 | 0.56 | 0.53 | 0.56 |
| 6 | 0.43 | 0.49 | 0.52 | 0.43 | 0.46 | 0.51 | 0.36 | 0.49 | 0.50 |

**The data in Fig. 10**

| Method | (a) | | (b) | | (c) | |
| --- | --- | --- | --- | --- | --- | --- |
|  | *FC* ($/hr) | *ET* (ton/h) | *FC* ($/hr) | *ET* (ton/h) | *FC* ($/hr) | *ET* (ton/h) |
| PSO | 611.31 | 0.24 | 649.24 | 0.21 | 625.71 | 0.22 |
| CSA | 610.62 | 0.23 | 656.87 | 0.20 | 626.10 | 0.21 |
| LIM | 611.02 | 0.20 | 644.98 | 0.20 | 625.29 | 0.21 |
| FA | 610.97 | 0.20 | 645.24 | 0.21 | 625.93 | 0.21 |
| ALO | 605.32 | 0.22 | 639.87 | 0.20 | 627.52 | 0.20 |
| IALO | 604.95 | 0.22 | 645.64 | 0.12 | 625.01 | 0.20 |

**The data in Fig. 11**

| Unit | ALO | | | PSO | | | CSA | | |
| --- | --- | --- | --- | --- | --- | --- | --- | --- | --- |
|  | (a) | (b) | (c) | (a) | (b) | (c) | (a) | (b) | (c) |
| 1 | 0.07 | 0.50 | 0.27 | 0.02 | 0.37 | 0.15 | 0.07 | 0.44 | 0.54 |
| 2 | 0.42 | 0.44 | 0.43 | 0.38 | 0.38 | 0.36 | 0.42 | 0.47 | 0.46 |
| 3 | 0.45 | 0.52 | 0.47 | 0.45 | 0.48 | 0.69 | 0.45 | 0.54 | 0.48 |
| 4 | 1.10 | 0.37 | 0.67 | 0.84 | 0.48 | 0.82 | 0.85 | 0.36 | 0.67 |
| 5 | 0.56 | 0.52 | 0.56 | 0.62 | 0.52 | 0.47 | 0.63 | 0.53 | 0.45 |
| 6 | 0.38 | 0.45 | 0.54 | 0.42 | 0.52 | 0.40 | 0.42 | 0.46 | 0.39 |
| Unit | LIM | | | FA | | | IALO | | |
|  | (a) | (b) | (c) | (a) | (b) | (c) | (a) | (b) | (c) |
| 1 | 0.07 | 0.41 | 0.55 | 0.07 | 0.38 | 0.54 | 0.07 | 0.41 | 0.46 |
| 2 | 0.12 | 0.36 | 0.53 | 0.55 | 0.40 | 0.46 | 0.42 | 0.45 | 0.43 |
| 3 | 0.65 | 0.36 | 0.59 | 0.45 | 0.33 | 0.48 | 0.65 | 0.53 | 0.47 |
| 4 | 0.90 | 0.47 | 0.33 | 1.07 | 0.47 | 0.67 | 1.06 | 0.39 | 0.67 |
| 5 | 0.56 | 0.43 | 0.57 | 0.64 | 0.50 | 0.56 | 0.56 | 0.52 | 0.55 |
| 6 | 0.40 | 0.50 | 0.57 | 0.42 | 0.54 | 0.41 | 0.28 | 0.46 | 0.40 |

**The data in Fig. 12**

| Target | PSO | CSA | LIM | FA | ALO | IALO |
| --- | --- | --- | --- | --- | --- | --- |
| Optimal cost | 1763.21 | 1770.14 | 1737.28 | 1760.03 | 1753.17 | 1742.33 |
| Optimal emissions | 1723.44 | 1726.18 | 1725.03 | 1715.26 | 1728.43 | 1709.12 |
| Best compromise | 1683.00 | 1556.14 | 1554.85 | 1632.08 | 1554.11 | 1542.54 |
